# Association of Metabolic Signatures with Nonalcoholic Fatty Liver Disease in Pediatric Population

**DOI:** 10.3390/metabo12090881

**Published:** 2022-09-19

**Authors:** Woori Chae, Kyung Jae Lee, Ki Young Huh, Jin Soo Moon, Jae Sung Ko, Joo-Youn Cho

**Affiliations:** 1 Department of Clinical Pharmacology and Therapeutics, Seoul National University College of Medicine and Hospital, Seoul 03080, Korea; 2 Department of Biomedical Sciences, Seoul National University College of Medicine, Seoul 03080, Korea; 3 Department of Pediatrics, Hallym University Sacred Heart Hospital, Anyang 14068, Korea; 4 Department of Pediatrics, Seoul National University College of Medicine, Seoul 03080, Korea

**Keywords:** nonalcoholic fatty liver disease, hepatic steatosis, pediatric obesity, plasma metabolomics, machine learning

## Abstract

Several adult omics studies have been conducted to understand the pathophysiology of nonalcoholic fatty liver disease (NAFLD). However, the histological features of children are different from those of adults, and the onset and progression of pediatric NAFLD are not fully understood. In this study, we aimed to evaluate the metabolome profile and metabolic pathway changes associated with pediatric NAFLD to elucidate its pathophysiology and to develop machine learning-based NAFLD diagnostic models. We analyzed the metabolic profiles of healthy control, lean NAFLD, overweight control, and overweight NAFLD groups of children and adolescent participants (*N* = 165) by assessing plasma samples. Additionally, we constructed diagnostic models by applying three machine learning methods (ElasticNet, random forest, and XGBoost) and multiple logistic regression by using NAFLD-specific metabolic features, genetic variants, and clinical data. We identified 18 NAFLD-specific metabolic features and metabolic changes in lipid, glutathione-related amino acid, and branched-chain amino acid metabolism by comparing the control and NAFLD groups in the overweight pediatric population. Additionally, we successfully developed and cross-validated diagnostic models that showed excellent diagnostic performance (ElasticNet and random forest model: area under the receiver operating characteristic curve, 0.95). Metabolome changes in the plasma of pediatric patients with NAFLD are associated with the pathophysiology of the disease and can be utilized as a less-invasive approach to diagnosing the disease.

## 1. Introduction

Nonalcoholic fatty liver disease (NAFLD) is one of the most frequent hepatic disorders in both adults and children [1,2]. Despite the increasing prevalence of NAFLD worldwide over the last few decades, no licensed drugs have been approved for its treatment. The difficulties in developing a single effective drug for NAFLD may be attributable to the complex pathophysiology of this disease. The risk factors for NAFLD include dietary, environmental, and genetic factors which show complex interactions resulting in insulin resistance and obesity [3]. These factors cause hepatic triglyceride accumulation, lipotoxicity due to the high levels of free fatty acids, and oxidative stress, which are involved in hepatic inflammation.

Contrary to adults, pediatric NAFLD presents unique histologic features, including moderate-to-severe steatosis and portal and periportal inflammation and fibrosis [4]. In addition, the early presentation of pediatric NAFLD suggests that it has a different etiology and pathophysiology than adult NAFLD. Specifically, pediatric NAFLD shows greater vulnerability to genetic and environmental factors [5,6]. For this reason, a thorough understanding of the distinctive characteristics of pediatric NAFLD is required to establish an appropriate treatment strategy in comparison with adult NAFLD. However, many aspects of pediatric NAFLD have not been elucidated, especially those related to the onset of NAFLD and liver damage during the disease’s progression in the pediatric population.

Recently, omics studies have been actively conducted to investigate and predict NAFLD and its severity by identifying the metabolic signatures that reflect the pathological status of the disease (i.e., the significant effect of genetic variants and metabolic changes) [7,8,9]. Some metabolomic studies have also reported metabolic signatures based on elements such as branched-chain amino acids (BCAAs), aromatic amino acids, and other lipidomic profiles known to be associated with NAFLD; however, most of these studies were conducted on adults and obese populations [10,11,12]. Furthermore, to the best of our knowledge, there have been no attempts to establish a diagnostic model that combines genetics, metabolomics, and clinical profiles in the pediatric population.

Based on our previous results that suggested the genetic effects of phospholipase-containing domain 3 (*PNPLA3*) rs738409, transmembrane 6 superfamily member 2 (*TM6SF2*) rs58542926, and the sorting and assembly machinery component 50 homolog (*SAMM50*) rs2073080 and rs3761472 on the development and severity of pediatric NAFLD were greater in the overweight group than the normal-weight group [13], we sought to find metabolic signatures associated with pediatric NAFLD in a subpopulation of the genetics study. In this study, we aimed to identify NAFLD-specific metabolic features and associated pathways by investigating the plasma metabolome signatures in pediatric patients with overweight NAFLD and comparing the signatures with a normal-weight population. In addition, by combining these metabolic features, genetic variations, and clinical data, we attempted to develop a novel diagnostic model of pediatric NAFLD.

## 2. Materials and Methods

### 2.1. Study Population

**Figure 1:**
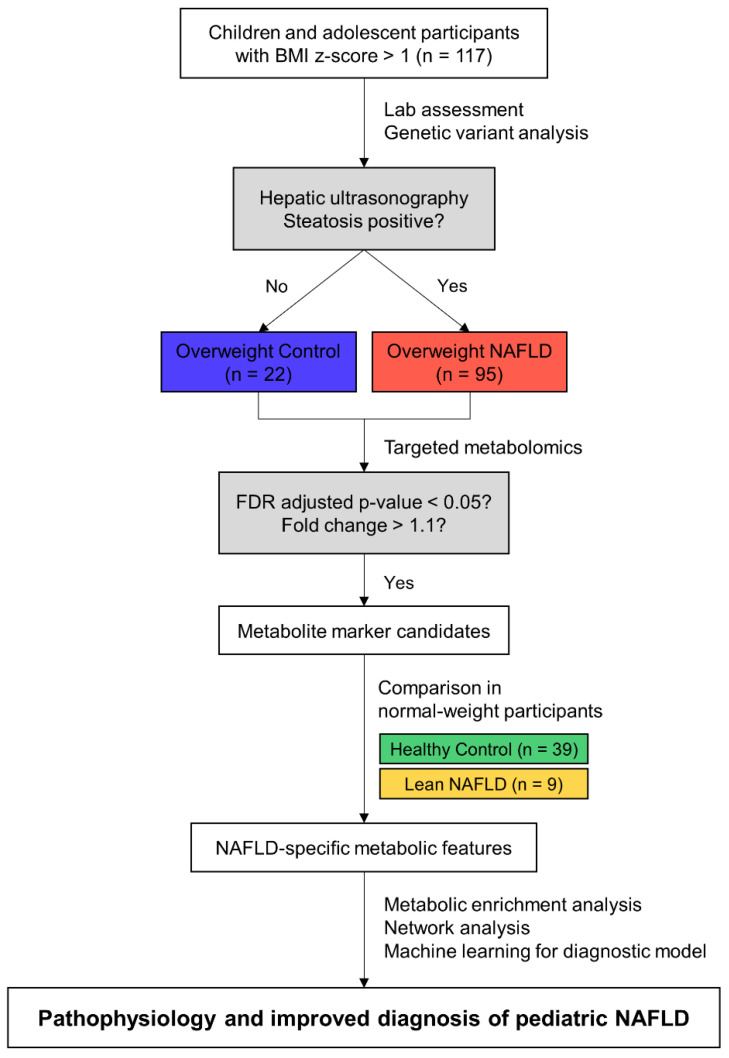
Schematic study design and analysis workflow. Abbreviations: BMI, body mass index; FDR, false discovery rate.

This study was approved by the Institutional Review Board (IRB) of each hospital (IRB No. 2018-10-015 by the Hallym University Sacred Heart Hospital and 1811-149-98 by Seoul National University Children’s Hospital) and conducted in accordance with the Declaration of Helsinki. We recruited children and adolescent participants who visited the pediatric departments of the Hallym University Sacred Heart Hospital and Seoul National University Children’s Hospital from January 2019 to May 2020, after obtaining informed consent from the children and their parents. We evaluated the presence and grade of fatty liver by ultrasonography. The grade of steatosis was evaluated as follows by comparing hepatic echogenicity to kidney parenchyma: normal, 0; mild, 1; moderate, 2; and severe, 3 [14,15]. The participants were categorized into four groups according to the steatosis grade determined by abdominal ultrasonography and body mass index (BMI) z-score based on the 2017 Korean National Growth Chart for children and adolescents [16]: healthy control (HC), steatosis grade = 0 and BMI z-score ≤ 1; lean NAFLD (LN), steatosis grade ≥ 1 and BMI z-score ≤ 1; overweight control (OC), steatosis grade = 0 and BMI z-score > 1; and overweight NAFLD (ON), steatosis grade ≥ 1 and BMI z-score > 1. We excluded participants who were taking alcohol or medications known to affect the results of liver function tests. Participants with viral hepatitis, such as hepatitis A, B, or C, or with Epstein–Barr virus, Wilson’s disease, autoimmune hepatitis, or muscular disease were also excluded. Thus, 165 subjects were included in this study. Figure 1 summarizes the schematic study design and analysis workflow to identify NAFLD-specific metabolic features. Note that we screened metabolite marker candidates in the overweight group and then verified them in the normal-weight group, considering obesity as a major risk factor for NAFLD and a confounding factor of plasma metabolome, as described in Figure S1.

### 2.2. Demographic and Laboratory Assessments

Anthropometric characteristics such as height, weight, and BMI z-score were evaluated. After overnight fasting, 4-mL peripheral blood samples were collected to assess the levels of insulin, hemoglobin A1c, and platelet count. In addition, serum samples were also collected by clotting blood for 30 min, followed by centrifugation at 4 °C. Laboratory assessments, including the measurement of the serum levels for fasting glucose, triglycerides, and cholesterol, were performed, and liver function tests, including measurements of serum aspartate transaminase (AST), alanine transaminase (ALT), gamma-glutamyl transferase (GGT), and alkaline phosphatase (ALP) activities, were also performed. The homeostatic model assessment for insulin resistance (HOMA-IR) was calculated as fasting glucose (mg/dL) multiplied by fasting insulin (mU/L) and then divided by 405. For clinical variables with missing data, such as GGT, fasting glucose, insulin, HbA1c, and HOMA-IR, appropriate statistical methods were chosen according to the number of data points for each variable following the exclusion of missing values.

### 2.3. Targeted Metabolomics in Plasma

For metabolomic analysis, 4 mL of blood was collected from each participant after overnight fasting and centrifuged at 4 °C. Separated plasma samples were collected and stored until use at −80 °C. Plasma metabolites, including 21 amino acids, 21 biogenic amines, 55 acylcarnitines (ACs), 18 diglycerides (DGs), 42 triglycerides (TGs), 172 phosphatidylcholines (PCs), 24 lysophosphatidylcholines (LPCs), 31 sphingomyelins (SMs), 9 ceramides (Cers), and 14 cholesteryl esters (CEs) and hexoses were analyzed using the AbsoluteIDQ^TM^ p400 HR kit (Biocrates Life Sciences AG, Innsbruck, Austria). Samples and reagents were prepared according to the manufacturer’s instructions. Additionally, we added three pooled plasma samples per plate for quality control and prepared them in the same way as the analytical samples to normalize the batch-to-batch effect. Briefly, frozen plasma samples were thawed on ice and vortexed, followed by centrifugation at 2750× *g*, 4 °C for 5 min before the samples were loaded onto a 96-well plate with a filter. After the addition of 10 μL of analytical and pooled plasma samples and calibration standards to each well, the plates were dried with a nitrogen evaporator and derivatized with phenyl isothiocyanate. Then, dried samples were extracted with an ammonium acetate solution in methanol and aliquoted into two deep-well plates for liquid chromatography mode and flow injection analysis (FIA) mode (described in the manual), followed by dilution with water and an FIA solvent, respectively. Both deep-well plates were placed in an autosampler of Ultimate 3000 ultra-performance liquid chromatography coupled with a Q Exactive Plus hybrid quadrupole-orbitrap mass spectrometer (Thermo Fisher Scientific, Waltham, MA, USA) and analyzed using the validated method.

Raw data were processed using the Xcalibur Software (Thermo Fisher Scientific, Waltham, MA, USA) and MetIDQ Oxygen (Biocrates Life Sciences AG, Innsbruck, Austria) to calculate the metabolite concentrations in each sample. The final quantitative results were exported micromolar values with pooled quality control normalization by the median. Subsequently, values under the lower limit of detection were imputed by one-fifth of the minimum positive values of their corresponding variables. These data were used for further analysis.

### 2.4. Statistical Analyses and Data Visualization

Comparison of the anthropometric and laboratory data between study groups was performed by a Kruskal–Wallis test, followed by a post-hoc Dunn’s multiple comparisons test with Prism 7 (GraphPad Software, San Diego, CA, USA). NAFLD-specific metabolic features (significant metabolites between OC and ON) were illustrated by a volcano plot. Significance was defined as a false discovery rate (FDR)-adjusted *p*-value < 0.05 and a fold change > 1.1, and calculated with MetaboAnalyst 5.0 [17]. Concentrations of significant metabolites between OC and ON were standardized by the autoscaling of features, followed by hierarchical metabolite clustering using the Ward method and Euclidean distance. The significance of NAFLD-specific metabolic features in the four groups was calculated by the Kruskal–Wallis test, followed by the two-stage step-up method proposed by Benjamini, Krieger, and Yekutieli to correct multiple comparisons by controlling FDR [18], in which the number of multiple comparisons per metabolites was four (HC vs. OC, HC vs. LN, OC vs. ON, and LN vs. ON). Correlation coefficients and two-tailed *p*-values of the NAFLD-specific metabolic features and HOMA-IR were determined by Spearman correlation analyses. Enriched metabolite sets between the OC and ON groups were identified by querying significant metabolites in an SMPDB-based database provided by MetaboAnalyst 5.0. Significant metabolites without a Human Metabolite Database (HMDB) ID or PubChem CID were excluded in this analysis. Chemical and biochemical relationships of significant metabolites were conceived by mapping onto MetaMapp [19] and by visualization with Cytoscape 3.8.2 [20].

### 2.5. Development of Diagnostic Models for NAFLD

Diagnostic models for NAFLD were developed and validated using machine learning techniques. The total dataset was split into training/validation and test datasets at a ratio of 4:1 and repeated 100 times (nested cross-validation). Additionally, each step was repeated three times for recurrent cross-validation. Variables were separated into NAFLD-specific metabolic features and clinical and genetic variables including age, sex, BMI z-score, AST, ALT, GGT, ALP, and three significant genetic variants (*PNPLA3* rs738409, *SAMM50* rs2073080, and rs3761472) (Supplemental Method S1). The diagnostic models using NAFLD-specific metabolic features were not adjusted for clinical factors during their development, as we wanted to create models that do not require the input of any clinical factors and can be compared with models using clinical and genetic variables. The following four machine learning models, which were previously used in the diagnosis of NAFLD, were evaluated [21]: logistic regression, the generalized linear model with an elastic net penalty (ElasticNet) [22], random forest [23], and extreme gradient boosting (XGBoost) [24]. The model hyperparameters, except the logistic regression model, were tuned using grid searching. The model performance for each repeated test set was evaluated by measuring the area under the receiver operating characteristic curve (AUROC), accuracy, sensitivity, specificity, and F1 score on the test. For the logistic regression model with a median AUROC, the regression coefficient, standard error, and z- and *p*-values of the selected variables were obtained. For ElasticNet, random forest, and XGBoost models which had a median AUROC, the variable importance scores of 18 NAFLD-specific metabolic features were calculated. Model building and validation were conducted using R version 4.1.0 [25] and R package *caret* [26].

## 3. Results

### 3.1. Clinical Characteristics of the Study Population

We performed plasma metabolomics on a pediatric cohort with NAFLD to understand the characteristics of pediatric NAFLD. In this study, 165 Korean children and adolescent participants aged 6 to 19 years were selected from the cohort. Their demographic features, including age, sex, BMI z-score, steatosis grade, liver function test results, and insulin resistance-related parameters, are summarized in Table 1 and Figure S2. As described in the methods section, steatosis grade and BMI z-score were used to classify the participants into four groups. The AST, ALT, and GGT levels were abnormally elevated in the NAFLD group, whereas those in the control group were in the normal range [27]. In contrast, the between-group difference in the ALP level was not significant. While insulin and HOMA-IR levels were significantly increased in the ON group compared to the OC group, no differences in HbA1c levels were observed between the HC, LN, OC, and ON groups.

### 3.2. Plasma Metabolic Profiles and Significant Metabolites between the Control and NAFLD Group in the Overweight Population

**Figure 2:**
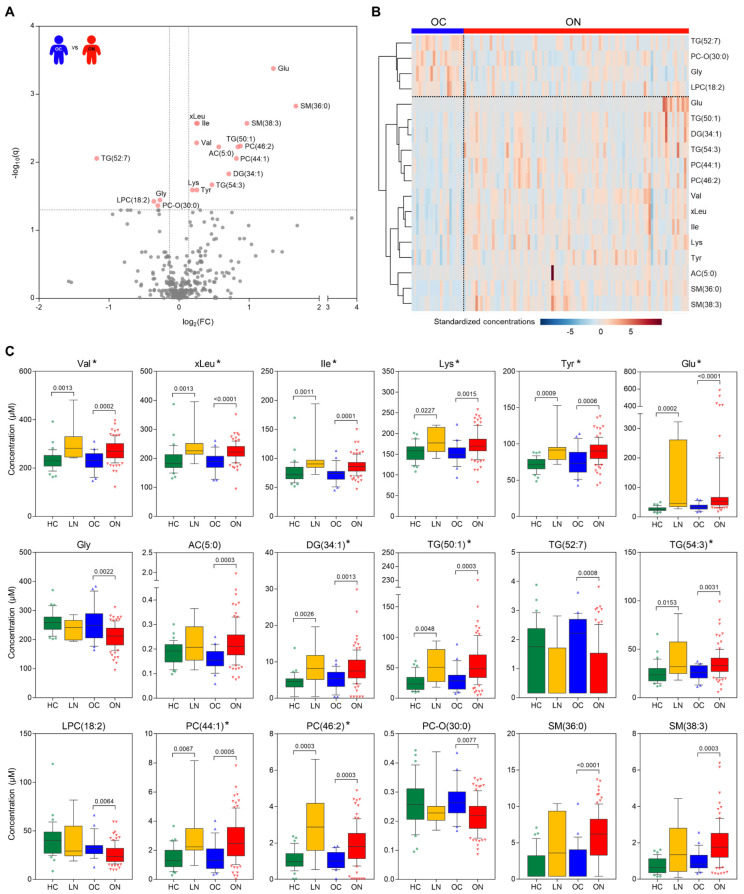
Metabolic features of overweight NAFLD (ON) compared to those of overweight control (OC). (**A**) A volcano plot and (**B**) a heat map of significant plasma metabolites (NAFLD-specific metabolic features, FDR adjusted *p*-value < 0.05 by Wilcoxon rank-sum test, fold change > 1.1) between OC and ON groups. In the volcano plot, significant metabolites are labeled with coral red. In the heat map, concentrations of each metabolite were standardized by the autoscaling of features, followed by metabolite clustering using the Ward method with Euclidean distance. (**C**) Concentrations of NAFLD-specific metabolic features in four groups. The box-and-whiskers plots show the median with the whiskers of the 10th and 90th percentile, while the X-axis represents the study groups. Significance (*q*-value) was calculated by the Kruskal–Wallis test followed by the two-stage step-up method of Benjamini, Krieger, and Yekutieli to correct for multiple comparisons by controlling FDR. The number of multiple comparisons per metabolite was four (HC vs. OC, HC vs. LN, OC vs. ON, and LN vs. ON). *q*-values < 0.05 were denoted in the panels. Metabolites were ordered according to their structural classes: BCAAs (Val, xLeu, and Ile), other amino acids (Lys, Tyr, Glu, and Gly), acylcarnitines and glycerolipids (AC (5:0), DG (34:1), TG (50:1), TG (52:7), and TG (54:3)), phosphatidylcholines (LPC (18:2), PC (44:1), PC (46:2), and PC-O (30:0)), and sphingomyelins (SM (36:0) and SM (38:3)). Metabolites marked with an asterisk (*) showed statistically significant differences in HC vs. LN, with the same direction of change as observed in the overweight population. Abbreviations: NAFLD, nonalcoholic fatty liver disease; FDR, false discovery rate; HC, healthy control; and LN, lean NAFLD.

We investigated the endogenous metabolic differences between the study groups by evaluating plasma samples with a targeted quantitation method. With targeted quantitation, we monitored a total of 408 metabolites and reliably detected 342 metabolites in the plasma. The metabolic distribution of the study population showed that the NAFLD groups (LN and ON) had relatively high intra-group variability compared to the control groups (HC and OC) (Figure S3). In total, 18 metabolites were significantly different (FDR-adjusted *p*-value < 0.05, fold change > 1.1, 14 up and 4 down) between the OC and ON groups (Figure 2A). In detail, the levels of multiple amino acids, including BCAAs (valine, leucine, and isoleucine), lysine, tyrosine, and glutamic acid, were significantly higher in the ON group than in the OC group, whereas the glycine level was lower in the ON group (Figure 2B). The levels of glycerolipids, phospholipids, and sphingolipids including TG (50:1), TG (54:3), DG (34:1), PC (46:2), PC (44:1), SM (36:0), and SM (38:3) were also elevated in the ON group, while TG (52:7), LPC (18:2), and PC-O (30:0) were reduced. Additionally, the valerylcarnitine (AC (5:0)) level was higher in the ON than in the OC group. In total, 11 of these 18 metabolites showed statistically significant differences, with the same direction of change as observed in the overweight population (Figure 2C, metabolites marked with an asterisk). The other 7 metabolites (Figure 2C, metabolites without an asterisk) also showed the same direction of change, although changes were not significant between the HC and LN groups due to the small number of participants. In addition, we performed regression analyses for each of the 18 metabolites, with the BMI z-score as a confounding factor, to investigate the effect of BMI and found that 16 of the metabolites, excluding AC (5:0) and glutamate, were significantly different between the OC and ON groups, even after controlling for the false-discovery rate (Benjamini–Hochberg method) (Table S1).

### 3.3. Correlation of Metabolic Features and Insulin Resistance

To demonstrate whether these significant metabolites are specifically correlated to NAFLD, we compared the metabolic features to clinical characteristics. We found that 13 of the 18 significant metabolites showed weak correlations with insulin resistance (Figure S4, Spearman r > 0.35, *p* < 0.05). However, no difference in HOMA-IR level was observed between the HC and the LN groups in the normal-weight population, whereas a higher HOMA-IR level was observed in the ON group than in the OC group (*p* = 0.0035 by post-hoc Dunn’s multiple comparison test following the Kruskal–Wallis test). Therefore, we regarded these 18 metabolites as “NAFLD-specific” metabolic features.

### 3.4. Relevance of NAFLD-Specific Metabolic Features in Metabolic Pathways

**Figure 3:**
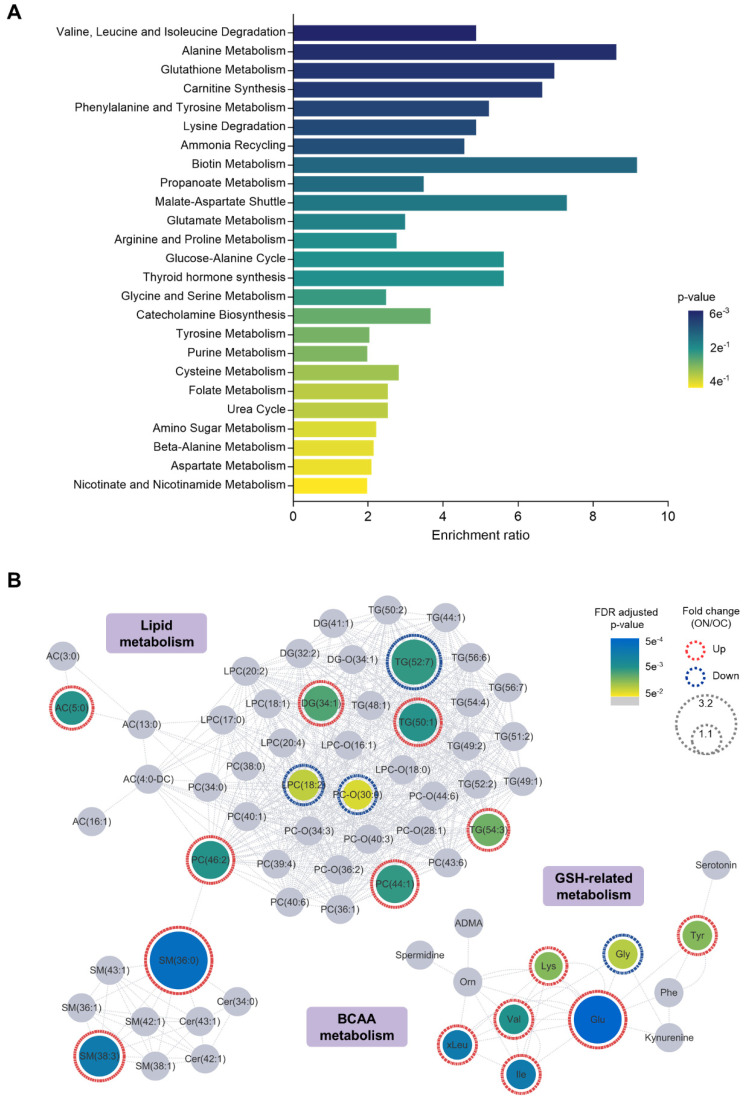
Relevance of NAFLD-specific metabolic features in metabolic pathways. (**A**) Enriched metabolite sets of NAFLD-specific metabolic features and (**B**) biochemical and chemical networks of the selected significant metabolites indicated the pertinent metabolic pathways in NAFLD status, such as branched-chain amino acid (BCAA), glutathione (GSH)-related, and lipid metabolism. In the network, the gray nodes indicate metabolites with raw *p*-values < 0.05 that were not significant after FDR adjustment. Abbreviations: NAFLD, nonalcoholic fatty liver disease; and FDR, false discovery rate.

Metabolite set enrichment analysis based on SMPDB was performed with the metabolic features to identify the metabolic pathways dysregulated by NAFLD. Several metabolite sets, including valine, leucine, and isoleucine degradation, alanine metabolism, glutathione metabolism, and carnitine synthesis were altered (enrichment ratio > 4, raw *p* < 0.05) in the ON group in comparison with the OC group (Figure 3A and Table S2). Next, we mapped NAFLD-specific metabolic features and other selected metabolites based on MetaMapp and visualized the network to explore the biochemical and chemical relationships of the features (Figure 3B). In this network, nodes with gradient color by FDR-adjusted *p*-value are NAFLD-specific metabolic features. Gray nodes indicate other selected metabolites with a raw *p*-value < 0.05 but no significance after FDR adjustment. We observed that the network can be divided into three main clusters: (1) lipids, (2) glutathione metabolism-related, and (3) BCAA-related metabolites. Most of the metabolic lipid biomarkers, including phosphatidylcholines, sphingomyelins, triglycerides, and diglycerides, were upregulated in the plasma of ON patients. Interestingly, upregulation of glutamic acid and tyrosine and downregulation of glycine, which are key metabolites in glutathione metabolism, were observed in ON patients. Moreover, plasma BCAAs, which are reported to be altered in adult NAFLD, were also upregulated in pediatric NAFLD.

### 3.5. Application of the Metabolic Features to Develop Diagnostic Models for Overweight NAFLD

**Figure 4:**
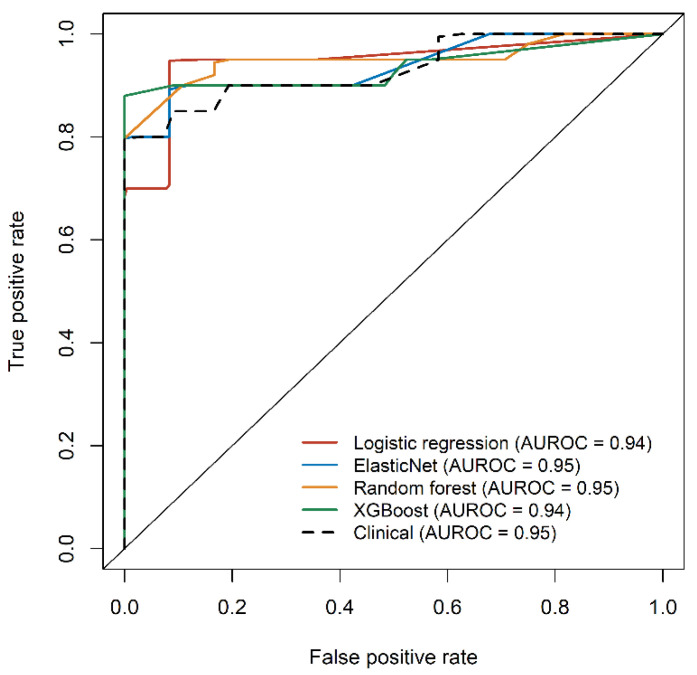
Receiver operating characteristic (ROC) analysis showed that NAFLD-specific metabolic features can be applied as important variables for NAFLD diagnostic model developed by machine learning approaches. The solid black line represents the line of unity. The area under the ROC curve (AUROC) values present the median values obtained from 100 repeated runs. Abbreviations: NAFLD, nonalcoholic fatty liver disease.

To suggest a pathophysiology-based complementary method for biopsy-proven diagnosis, we established diagnostic models based on machine learning approaches using the 18 NAFLD-specific metabolic features found in this study. (Table S3). Based on coefficients of the logistic regression model and variable importance scores in other models, the following metabolites were identified as significant features: valine, tyrosine, glutamic acid, glycine, and SM (38:3) (Tables S4 and S5). All four diagnostic models using NAFLD-specific metabolic features demonstrated excellent predictive performances, with median AUROC values of 0.95 (ElasticNet and random forest) and 0.94 (logistic regression and XGBoost) without significant differences between the models (Figure 4, colored line).

We also developed a logistic regression model using clinical and genetic variables. As the number of risk alleles of rs738409 (PNPLA3), rs2073080 (SAMM50), and rs3761472 (SAMM50) was positively associated with the presence of NAFLD (Table S6, *p* = 0.0029, 0.0011, 0.0004 for Cochran–Armitage, 0.0019, 0.0030, 0.0005 for Chi-squared test), and the proportions of homozygous risk alleles were significantly higher in the NAFLD group than in the control group, these three variants were selected as the genetic variables. The model also showed comparable performance (Figure 4, black dashed line) to the metabolic feature-based models, with the BMI z-score and ALT levels having a critical influence on the model. Among the diagnostic models using NAFLD-specific metabolic features, the ElasticNet model outperformed the other models with the highest median AUROC, yielding a sensitivity of 0.75 and specificity of 0.95.

## 4. Discussion

**Figure 5:**
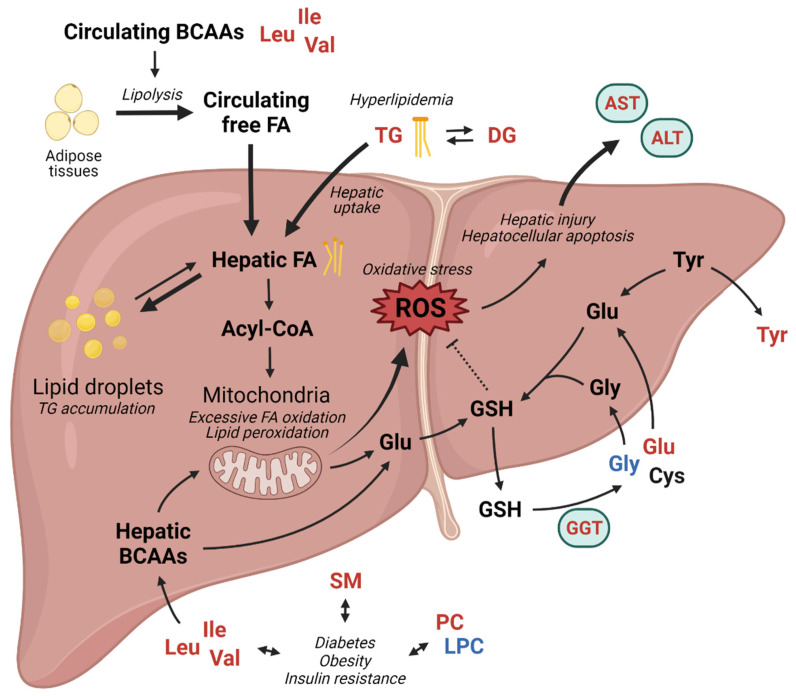
Alterations of metabolic pathways reflecting pathophysiology of NAFLD in overweight pediatric patients. Red: increased level or activity in NAFLD group; Blue: decreased level or activity in NAFLD group. Created with BioRender.com. Abbreviations: NAFLD, nonalcoholic fatty liver disease; BCAAs, branched-chain amino acids; Leu, leucine; Ile, isoleucine; Val, valine; FA, fatty acid; TG, triglyceride; DG, diglyceride; AST, aspartate aminotransferase; ALT, alanine aminotransferase; ROS, reactive oxygen species; Glu, glutamic acid; GSH, glutathione; GGT, gamma-glutamyl transferase; Gly, glycine; Tyr, tyrosine; Cys, cysteine; SM, sphingomyelin; PC, phosphatidylcholine; and LPC, lysophosphatidylcholine.

In this study, plasma metabolomic data revealed that in the diseased state, circulating metabolite levels related to glutathione-related amino acid metabolism, lipid metabolism, and BCAA metabolism were remarkably altered. On the basis of these results, we propose the potential effects of altered metabolisms on NAFLD in pediatric patients in Figure 5.

Glutathione metabolism-related metabolite levels, including those of glutamate and glycine, were significantly changed in the blood and may be important therapeutic targets, as excessive ROS-induced oxidative stress affects the progression of NAFLD. Increased circulating glutamic acid and decreased glycine levels in NALFD have been consistently reported in both pediatric [28] and adult populations [10,29,30,31] in addition to our results; however, the causes for their changes remain unclear. Interestingly, Oren et al. suggested that impaired glycine metabolism might play a causative role in NAFLD [32]. Several clinical studies have been conducted to evaluate the effect of glycine supplementation [33,34,35], as glycine is a limiting substrate of the de novo synthesis of endogenous glutathione [31], which may have therapeutic potential. Moreover, White et al. observed that the impairment of BCAA metabolism in obesity can also affect the decreased level of circulating glycine [36].

The elevated blood levels of DG and TG in patients with NAFLD shown in this study may also be associated with increased oxidative stress and lipid peroxidation in hepatocytes. Once circulating DGs and TGs are transferred by hepatic uptake, they can accumulate as lipid droplets or convert into free fatty acids in the liver. The induction of oxidative stress and lipid peroxidation by the mitochondrial oxidation of excessive hepatic free fatty acids resulted in hepatocellular apoptosis [37,38], which was reflected in the markedly increased serum AST and ALT levels of the NAFLD groups in this study. Similarly, modifications in sphingolipid and phospholipid metabolism are also associated with metabolic disease and NAFLD [39,40,41,42]. In young adults with obesity, serum SMs with saturated acyl chains have been reported to be associated with obesity, insulin resistance, and decreased liver function [43]. Some lipidomic studies have suggested that the plasma PC/PE ratio is associated with obesity [44]. However, the mechanisms linking SMs, PCs, and LPCs with liver steatosis and NAFLD are still unclear [40] and there is a lack of consistency in the level and pattern of the reported sphingolipids and phospholipids in both pediatric and adult NAFLD patients which cannot be explained by the currently available information in the literature.

The concentration of systemic BCAAs is altered in various metabolic diseases such as diabetes, insulin resistance, and obesity, which are well-known etiologic factors in NAFLD [45,46,47]. We observed elevated circulating BCAAs levels in the pediatric population with overweight NAFLD, which has been reported by several in vitro and in vivo studies in pediatric [12] and adult populations [48,49,50,51]. In addition to serving as substrates for protein synthesis and energy production, BCAAs also stimulate protein synthesis, inhibit proteolysis, and affect glucose metabolism and oxidative stress [47,52], indicating that the homeostatic regulation of BCAA levels is crucial to maintain physiological status. Excessive systemic levels of BCAAs can increase abnormal adipocyte lipolysis and suppress hepatic lipogenesis, resulting in hyperlipidemia and hepatic lipotoxicity [51], which is also supported by increased blood DGs and TGs in the overweight NAFLD group in this study. In accordance with the present results, BCAA-based metabolic signatures have been suggested to predict liver steatosis and NAFLD in children and adolescents with obesity [12,53].

Taken together, the findings of the present study have some major clinical implications. First, we investigated the metabolomic distributions of the HC, LN, OC, and ON groups and found that both the LN and ON groups showed metabolic heterogeneity, which may be closely related to the complex pathophysiology of NAFLD. We also identified metabolic changes to elucidate the characteristics and mechanisms of pediatric NAFLD and NAFLD-specific metabolic features by comparing the metabolomic signatures of the overweight and normal-weight groups. Additionally, we revealed that these metabolic changes, which can be observed in the adult population, emerged during the adolescent period. As most of the significant metabolites between the OC and ON groups, including BCAAs, are known to be related to insulin resistance and obesity, it is difficult to evaluate whether the significant metabolites are NAFLD-specific. Despite there being partially missing data, such as fasting insulin and HOMA-IR, we showed that these metabolic features are NAFLD-specific, regardless of insulin resistance and obesity. Additionally, using the NAFLD-specific metabolic features, we successfully suggested cross-validated diagnostic models based on pathophysiology that might be easily applied in clinical practice and showed better performance than other diagnostic models based on metabolomics [7,54,55]. These encouraging results demonstrate that the diagnosis of NAFLD with only a small volume of plasma may ameliorate the limitations of a liver biopsy and allow for the high-throughput screening of pediatric NAFLD, even in school. We also found that clinical features, such as the BMI z-score and liver function test results, as well as the metabolic features, were also directly associated with the development of NAFLD in pediatric patients, which is well reflected in the outstanding performance of the machine learning models using clinical and genetic variables. The fact that the increased level of liver function tests in adults resulted from complex interactions between the disease pathophysiology and external factors, such as smoking, alcohol, and stress, whereas those in children mainly resulted from hepatic inflammation itself, can be one of the explanations why the models using clinical and genetic variables developed in this pediatric population study show excellent performance, comparing with other diagnostic models that were previously reported in the adult population [8,54,56].

This study had some limitations, however. Although liver biopsies are deemed the gold standard for diagnosing liver steatosis and fibrosis, one of the most challenging issues in this study was obtaining liver biopsies from the study cohort. Due to the invasiveness and effectiveness of biopsies, and the ethical considerations related to performing biopsies in pediatric patients, we examined steatosis grades by using hepatic ultrasonography instead of liver biopsies. The targeted metabolomics used in this study effectively excluded exogenous compounds and unwanted analytical noise, however, this may only have allowed for a partial explanation of the issue in comparison with untargeted approaches, which implies there is a possibility of unrevealed metabolic signatures. In addition, the diagnostic models based on machine learning approaches in this study showed excellent performance; however, further evaluation of the models using external validation cohorts is required. Although the changes in the levels of circulating plasma metabolites directly reflect metabolic changes in the liver, the lack of observation of metabolic changes in the hepatocytes may have influenced the interpretation of the results. For example, the antioxidant effects of significant metabolic markers, or free fatty acid accumulation in the liver, could not be confirmed in this study. Lastly, a fact that should also be carefully considered before interpreting the results is that NAFLD had already occurred, making it difficult to assess whether the differences in the metabolite levels were a reflection of their etiologic roles, or if these were a consequence of the disease, as this causality problem is a common limitation of observational studies. Although the associations between NAFLD and plasma metabolites were observable in this study, further research efforts, such as in vitro/in vivo studies using cell lines or model organisms, are required to confirm the disease mechanism-related functions of selected metabolites and the direction of causality.

In summary, to answer questions regarding the characteristics of pediatric NAFLD, we explored metabolic profiles and found several significant alterations. This study demonstrated that dysregulated glutathione, lipid, and BCAA metabolism were linked to the pathophysiological conditions underlying NAFLD. Despite the restricted sample accessibility, these findings provide additional evidence for the pathophysiology of pediatric NAFLD that might be useful as potential therapeutic targets for new drug development and as ancillary diagnostic biomarkers to establish early strategies to alleviate NAFLD in pediatric patients.

## Figures and Tables

**Table 1:** Clinical characteristics of the study population according to the occurrence of obesity and NAFLD.

	Healthy Control (HC)	Lean NAFLD (LN)	Overweight Control (OC)	Overweight NAFLD (ON)	Significance *
The number of subjects	39	9	22	95	-
Age (year)	14.3 (8.7–18.6)	10.6 (9.6–17.4)	14.3 (6.6–17.6)	12.5 (6.4–18.9)	0.3546
Sex (male/female)	27/12	9/0	12/10	74/21	-
BMI z-score	−0.45 (−2.37–0.96)	0.88 (0.74–1.00)	1.63 (1.11–3.04)	2.42 (1.08–5.94)	<0.0001
Steatosis grade by ultrasonography	0 (0)	2 (1.5–2.5)	0 (0)	2 (1–3)	-
AST (IU/L)	21 {17–25}	51 {30–57}	20 {16–25}	46 {29–76}	<0.0001
ALT (IU/L)	12 {10–17}	73 {52–91}	18 {14–25}	84 {40–144}	<0.0001
GGT (IU/L)	11 {9–13}	28 {19–42}	16 {13–19}	34 {22–57} ^†^	<0.0001
ALP (IU/L)	202 {141–290}	267 {227–370}	137 {90–305}	256 {128–371}	0.0500
Fasting glucose (mg/dL)	97 {91–102}	96 {94–105}	101 {99–104}	100 {95–108} ^‡^	0.0175
Insulin (mU/L) ^§^	7.3 {4.5–16}	10.9 {8.1–48}	9.0 {6.3–12}	17.5 {12–23}	0.0012
HOMA-IR ^§^	1.74 {1.16–4.89}	2.48 {1.96–12.2}	2.35 {1.55–2.89}	4.27 {3.01–5.66}	0.0028
HbA1c (%) ^¶^	5.3 {4.8–5.9}	5.3 {5.0–5.8}	5.2 {5.0–5.4}	5.4 {5.1–5.7}	0.2264

* Significance by the Kruskal–Wallis test; ^†^ *n* = 93; ^‡^ *n* = 94; ^§^ HC (*n* = 4), LN (*n* = 5), OC (*n* = 12), ^¶^ ON (*n* = 67); HC (*n* = 4), LN (*n* = 3), OC (*n* = 15), and ON (*n* = 77). Continuous variables are given as the median (min-max) or median {25th–75th percentile}. Abbreviations: NAFLD, nonalcoholic fatty liver disease; BMI, body mass index; AST, aspartate aminotransferase; ALT, alanine aminotransferase; GGT, gamma-glutamyl transferase; and ALP, alkaline phosphatase.

## Data Availability

This data is available at the NIH Common Fund’s National Metabolomics Data Repository (NMDR) website, the Metabolomics Workbench, https://www.metabolomicsworkbench.org (accessed on 25 August 2022) where it has been assigned Project ID PR001451. The data can be accessed directly via its Project DOI: 10.21228/M85H8N. This work is supported by NIH grant U2C-DK119886.

## Supplementary Materials

The following supporting information can be downloaded at: https://www.mdpi.com/article/10.3390/metabo12090881/s1, Supplementary Method S1: Genotyping of NAFLD-related genetic variants; Table S1. Significant differences in 18 NAFLD-specific metabolic features after BMI z-score adjustment; Table S2. Enriched metabolite sets of significant metabolites in the overweight control and overweight NAFLD group based on SMPDB by metabolite set enrichment analysis; Table S3. Summary of the performance metrics from 100 repeated runs of the diagnostic model using four machine learning methods; Table S4. Multiple logistic regression model using NAFLD-specific metabolic features and clinical and genetic variables; Table S5. Variable importance of three diagnostic models using NAFLD-specific metabolic features; Table S6. Genotype frequencies of seven genetic variants of the study population; Figure S1. Differences in metabolic profiles of subgroups of the study population; Figure S2. Clinical characteristics of the study population according to the occurrence of obesity and NAFLD; Figure S3. Pareto-scaled score plot of principal component analysis showing metabolomic distribution in the study population (HC, LN, OC, and ON); Figure S4. Spearman correlations between NAFLD-specific metabolic features and insulin resistance with *p* < 0.05, except Tyr, Lys, Gly, LPC (18:2), and PC-O (30:0).
