# Supplementary material for: Association of Metabolic Signatures with Nonalcoholic Fatty Liver Disease in Pediatric Population
Source: Metabolites. 2022 Sep 19;12(9):881. doi: 10.3390/metabo12090881 (PMC9503976; doi:10.3390/metabo12090881)
Supplement: Supplementary file 1 [file metabolites-12-00881-s001.zip › metabolites-1907683-supplementary.pdf]

Supplementary Materials

Association of Metabolic Signatures with Nonalcoholic Fatty Liver Disease in Pediatric Population

Woori Chae [1,2,†], Kyung Jae Lee [3,†,‡], Ki Young Huh [1], Jin Soo Moon [4], Jae Sung Ko [4,*] and Joo-Youun Cho [1,2,*]

1 Department of Clinical Pharmacology and Therapeutics, Seoul National University College of Medicine and Hospital, Seoul 03080, Korea
2 Department of Biomedical Sciences, Seoul National University College of Medicine, Seoul 03080, Korea
3 Department of Pediatrics, Hallym University Sacred Heart Hospital, Anyang 14068, Korea
4 Department of Pediatrics, Seoul National University College of Medicine, Seoul 03080, Korea
* Correspondence: kojs@snu.ac.kr (J.S.K.); joocho@snu.ac.kr (J.-Y.C.); Tel.: +82-2-2072-2197 (J.S.K.); Tel.: +82-2-740-8286 (J.-Y.C.)
† These authors equally contributed to this work.
‡ Current Address: Department of Pediatrics, Seoul National University Children's Hospital, Seoul 03080, Korea.

Supplementary Method S1: Genotyping of NAFLD-related genetic variants

Genomic DNA was extracted from peripheral blood using a Gentra Puregene Blood Kit (Qiagen, Hilden, Germany) according to the manufacturer's instructions. Participants were genotyped for seven variants of six genes, including PNPLA3 rs738409, glucokinase regulator gene (GCKR) rs780094, apolipoprotein C3 (APOC3) rs2070666, SAMM50 rs2073080 and rs3761472, TM6SF2 rs58542926, and membrane-bound O-acyltransferase domain-containing 7 (MBOAT7) rs641738 by using the TaqMan allelic discrimination assay with an ABI 7900HT Real-Time PCR system (Applied Biosystems, Foster City, CA, USA). Predesigned assay primers and probes were obtained from Applied Biosystems.

List of Supplemental Tables and Figures

Table S1. Significant differences in 18 NAFLD-specific metabolic features after BMI z-score adjustment.

Table S2. Enriched metabolite sets of significant metabolites in the overweight control and overweight NAFLD group based on SMPDB by metabolite set enrichment analysis. Abbreviations: NAFLD, nonalcoholic fatty liver disease; FDR, false discovery rate.

Table S3. Summary of the performance metrics from 100 repeated runs of the diagnostic model using four machine learning methods.

Table S4. Multiple logistic regression model using NAFLD-specific metabolic features and clinical and genetic variables.

Table S5. Variable importance of three diagnostic models using NAFLD-specific metabolic features.

Table S6. Genotype frequencies of seven genetic variants of the study population.

Figure S1. Differences in metabolic profiles of subgroups of the study population.

Figure S2. Clinical characteristics of the study population according to the occurrence of obesity and NAFLD. Abbreviations: NAFLD, nonalcoholic fatty liver disease; BMI, body mass index; AST, aspartate aminotransferase; ALT, alanine aminotransferase; GGT, gamma-glutamyltransferase; ALP, alkaline phosphatase; HbA1c, Hemoglobin A1c; and HOMA-IR, homeostatic model assessment for insulin resistance.

Figure S3. Pareto-scaled score plot of principal component analysis showing metabolomic distribution in the study population (HC, LN, OC, and ON).

Figure S4. Spearman correlations between NAFLD-specific metabolic features and insulin resistance with $p < 0.05$, except Tyr, Lys, Gly, LPC (18:2), and PC-O (30:0). Abbreviations: Glu, glutamic acid; TG, triglyceride; DG, diglyceride; xLeu, sum of leucine and isoleucine levels; PC, phosphatidylcholine; SM, sphingomyelin; Ile, isoleucine; Val, valine; AC, acylcarnitine; Tyr, tyrosine; Lys, lysine; Gly, glycine; LPC, lysophosphatidylcholine; and PC-O, ether-linked phosphatidylcholine.

Table S1. Significant differences in 18 NAFLD-specific metabolic features after BMI z-score adjustment.

Metabolite	Raw p-value*	FDR**-adjusted p-value
AC (5:0)	0.12682	0.1268
Glu	0.10228	0.1083
Gly	0.00115	0.0036
Ile	0.00159	0.0036
Lys	0.01402	0.0168
Tyr	0.00157	0.0036
Val	0.00145	0.0036
xLeu	0.00058	0.0035
(34:1)	0.01595	0.0179
TG (50:1)	0.01134	0.0146
TG (52:7)	0.00119	0.0036
TG (54:3)	0.00810	0.0121
LPC (18:2)	0.00910	0.0126
PC (44:1)	0.00724	0.0118
PC (46:2)	0.00309	0.0056
PC-O (30:0)	0.00057	0.0035
SM (36:0)	0.00007	0.0013
SM (38:3)	0.00179	0.0036

* Raw p-values were calculated from multiple linear regression analyses (metabolite ~ BMI z-score + Phenotype (OC or ON)).
** False discovery rate (FDR) was controlled for using the Benjamini–Hochberg method.

Table S2. Enriched metabolite sets of significant metabolites in the overweight control and overweight NAFLD group based on SMPDB by metabolite set enrichment analysis. Abbreviations: NAFLD, nonalcoholic fatty liver disease; FDR, false discovery rate.

Metabolite sets	Total[†]	Expected hits	Observed hits	Enrichment ratio	Raw p	Holm p	FDR
Valine, Leucine, and Isoleucine Degradation	60	0.820	4	4.88	6.86E-03	0.672	0.672
Alanine Metabolism	17	0.232	2	8.62	2.10E-02	1	0.841
Glutathione Metabolism	21	0.287	2	6.97	3.14E-02	1	0.841
Carnitine Synthesis	22	0.301	2	6.64	3.43E-02	1	0.841
Phenylalanine and Tyrosine Metabolism	28	0.383	2	5.22	5.36E-02	1	0.954
Lysine Degradation	30	0.410	2	4.88	6.07E-02	1	0.954
Ammonia Recycling	32	0.438	2	4.57	6.81E-02	1	0.954
Biotin Metabolism	8	0.109	1	9.17	1.05E-01	1	1
Propanoate Metabolism	42	0.574	2	3.48	1.09E-01	1	1
Malate-Aspartate Shuttle	10	0.137	1	7.30	1.29E-01	1	1
Glutamate Metabolism	49	0.670	2	2.99	1.42E-01	1	1
Arginine and Proline Metabolism	53	0.725	2	2.76	1.61E-01	1	1
Glucose-Alanine Cycle	13	0.178	1	5.62	1.65E-01	1	1
Thyroid Hormone Synthesis	13	0.178	1	5.62	1.65E-01	1	1
Glycine and Serine Metabolism	59	0.807	2	2.48	1.91E-01	1	1
Catecholamine Biosynthesis	20	0.273	1	3.66	2.43E-01	1	1
Tyrosine Metabolism	72	0.984	2	2.03	2.58E-01	1	1
Purine Metabolism	74	1.010	2	1.98	2.68E-01	1	1
Cysteine Metabolism	26	0.355	1	2.82	3.04E-01	1	1
Folate Metabolism	29	0.396	1	2.53	3.33E-01	1	1
Urea Cycle	29	0.396	1	2.53	3.33E-01	1	1
Amino Sugar Metabolism	33	0.451	1	2.22	3.70E-01	1	1
Beta-Alanine Metabolism	34	0.465	1	2.15	3.79E-01	1	1
Aspartate Metabolism	35	0.479	1	2.09	3.87E-01	1	1
Nicotinate and Nicotinamide Metabolism	37	0.506	1	1.98	4.05E-01	1	1
Porphyrin Metabolism	40	0.547	1	1.83	4.30E-01	1	1
Methionine Metabolism	43	0.588	1	1.70	4.54E-01	1	1
Histidine Metabolism	43	0.588	1	1.70	4.54E-01	1	1
Warburg Effect	58	0.793	1	1.26	5.60E-01	1	1
Tryptophan Metabolism	60	0.820	1	1.22	5.73E-01	1	1

Bile Acid Biosynthesis	65	0.889	1	1.12	6.03E-01	1	1
Arachidonic Acid Metabolism	69	0.943	1	1.06	6.26E-01	1	1

† The number of metabolites in a metabolite set.

Table S3. Summary of the performance metrics from 100 repeated runs of the diagnostic model using four machine learning methods.

	NAFLD-specific metabolic features				Clinical and genetic variables			
	**Logistic regression**	**ElasticNet**	**Random forest**	**XGBoost**	**Logistic regression**	**ElasticNet**	**Random forest**	**XGBoost**
**AUROC**	0.94 (0.76-1.00)	0.95 (0.85-1.00)	0.95 (0.80-1.00)	0.94 (0.78-1.00)	0.95 (0.80-1.00)	0.95 (0.86-1.00)	0.96 (0.88-1.00)	0.95 (0.84-1.00)
**Accuracy**	0.88 (0.69-0.97)	0.88 (0.75-1.00)	0.88 (0.72-0.97)	0.84 (0.72-0.94)	0.88 (0.72-0.97)	0.88 (0.81-1.00)	0.88 (0.75-1.00)	0.88 (0.75-0.97)
**Sensitivity**	0.83 (0.50-1.00)	0.75 (0.58-1.00)	0.83 (0.50-1.00)	0.75 (0.42-1.00)	0.83 (0.58-1.00)	0.83 (0.50-1.00)	0.83 (0.50-1.00)	0.83 (0.50-1.00)
**Specificity**	0.90 (0.70-1.00)	0.95 (0.75-1.00)	0.90 (0.65-1.00)	0.90 (0.70-1.00)	0.90 (0.70-1.00)	0.95 (0.75-1.00)	0.90 (0.75-1.00)	0.90 (0.75-1.00)
**F1 score**	0.81 (0.55-0.96)	0.82 (0.67-1.00)	0.82 (0.63-0.96)	0.78 (0.53-0.92)	0.86 (0.67-0.96)	0.86 (0.67-1.00)	0.84 (0.63-1.00)	0.82 (0.60-0.96)

Values are given as median (minimum-maximum).

Table S4. Multiple logistic regression model using NAFLD-specific metabolic features and clinical and genetic variables.

Variables	Coefficient	SE	z-value	p-value
**NAFLD-specific metabolic features**				
(Intercept)	4.395	1.180	3.725	0.0002
Val	5.131	2.252	2.278	0.0227
Ile	-3.652	2.460	-1.485	0.1376
Lys	3.081	2.065	1.492	0.1356
Tyr	7.123	2.104	3.385	0.0007
Glu	28.869	9.109	3.169	0.0015
Gly	-4.840	1.896	-2.552	0.0107
TG (52:7)	-2.081	1.248	-1.668	0.0954
PC-O (30:0)	-2.910	1.527	-1.906	0.0566
SM (38:3)	5.852	2.637	2.220	0.0264
**Clinical and genetic variables**				
(Intercept)	3.374	0.975	3.460	0.0005
BMI z-score	7.379	1.833	4.027	< 0.0001
Sex (female)	-2.433	0.951	-2.560	0.0105
ALT	15.717	4.413	3.561	0.0004
PNPLA3 rs738409	2.585	1.105	2.339	0.0193

Table S5. Variable importance of three diagnostic models using NAFLD-specific metabolic features.

ElasticNet (glmnet)		Random forest (ranger)		XGBoost (xgbTree)	
**Metabolite**	**Score**	**Metabolite**	**Score**	**Metabolite**	**Score**
Tyr	100	SM(38:3)	100	Tyr	100
SM(38:3)	85.872	Tyr	85.905	SM(38:3)	71.746
Glu	78.406	xLeu	73.956	Gly	53.489
Gly	73.254	Val	73.506	Glu	44.524
PC:O(30:0)	63.572	SM(36:0)	63.616	Val	26.3
Val	54.924	Gly	48.47	PC:O(30:0)	21.545
AC(5:0)	53.196	Ile	43.735	xLeu	21.016
LPC(18:2)	52.006	LPC(18:2)	40.95	TG(52:7)	20.137
TG(50:1)	41.624	TG(50:1	37.987	PC(46:2)	18.839
SM(36:0)	35.029	Glu	32.414	Lys	16.355
PC(46:2)	34.573	PC(46:2)	28.593	LPC(18:2)	13.294
PC(44:1)	34.356	PC:O(30:0)	23.944	SM(36:0)	10.795
DG(34:1)	31.008	DG(34:1)	23.745	TG(50:1)	7.421
Ile	30.839	TG(54:3)	16.24	Ile	6.543
xLeu	23.012	PC(44:1)	15.903	PC(44:1)	4.163
TG(52:7)	15.234	AC(5:0)	10.108	DG(34:1)	1.194
TG(54:3)	7.187	Lys	6.875	TG(54:3)	1.164
Lys	0	TG(52:7)	0	AC(5:0)	0

Table S6. Genotype frequencies of seven genetic variants of the study population.

Gene	rs number	Transition	Genotype frequencies by the number of risk alleles of each group						p-value	
			Control			NAFLD			Cochran–Armitage	Chi-squared
			0	1	2	0	1	2		
PNPLA3	rs738409[†]	C>G[‡]	0.311	0.475	0.213	0.212	0.298	0.490	0.0029[§]	0.0019[§]
GCKR	rs780094	T[‡]>C	0.246	0.492	0.262	0.183	0.442	0.375	0.1315	0.2989
APOC3	rs2070666	T>A[‡]	0.508	0.410	0.082	0.462	0.433	0.106	0.5052	0.7998
SAMM50	rs2073080[†]	C>T[‡]	0.311	0.508	0.180	0.173	0.394	0.433	0.0011[§]	0.0030[§]
SAMM50	rs3761472[†]	A>G[‡]	0.311	0.541	0.148	0.183	0.375	0.442	0.0004[§]	0.0005[§]
TM6SF2	rs58542926	C>T[‡]	0.803	0.197	0	0.769	0.231	0	0.6092	NA
MBOAT7	rs641738	C>T[‡]	0.557	0.410	0.033	0.587	0.365	0.048	0.8816	0.7915

Allele frequencies and genotype frequencies were calculated according to the number of risk alleles of each genetic variant. An association between the presence of NAFLD and the number of risk alleles of each genetic variant was evaluated by the Cochran–Armitage test for trend and Chi-squared test with R version 4.1.0 (R Foundation for Statistical Computing, Vienna, Austria).

† These genetic variants showed linkage disequilibrium.

‡ Risk allele of the genetic variant.

§ Significant by Cochran–Armitage test or Chi-squared test ($p < 0.05$).

Figure S1. Differences in metabolic profiles of subgroups of the study population.

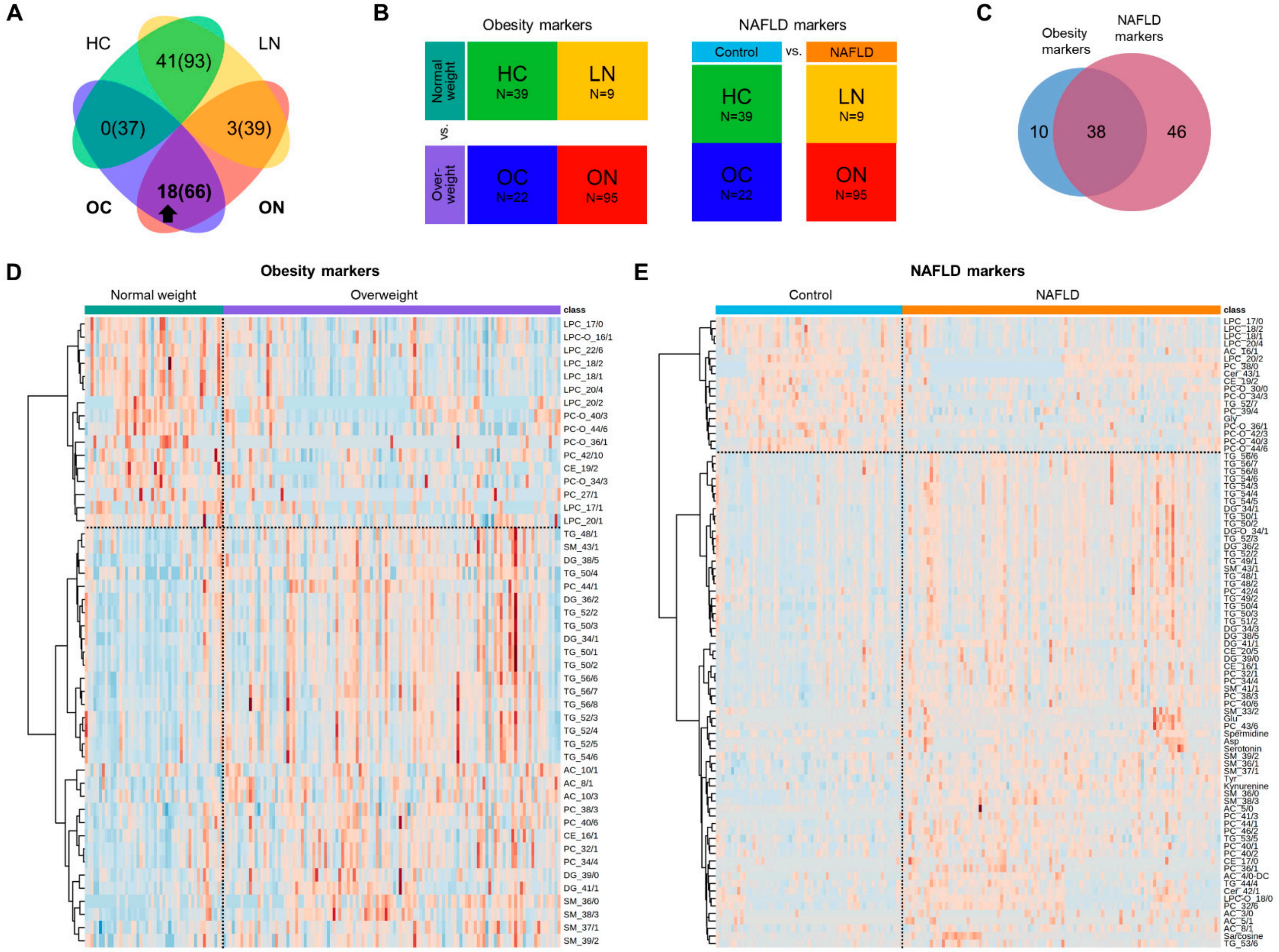

Comments on Figure S1: Figure S1A shows the number of significant metabolites (FDR-adjusted p-value < 0.05, fold change > 1.1) between HC and LN, HC and OC, LN and ON, or OC and ON by Wilcoxon's rank-sum test; the findings implied that more metabolites were changed by NAFLD than by obesity (none of the metabolites were even significantly changed in HC vs. OC). We also compared the metabolic profiles of subgroups, control versus NAFLD group, or normal-weight versus overweight group (Figure S1B). A greater number of significant metabolites (FDR-adjusted p-value < 0.05, fold change > 1.2) were observed in the comparison between the control and NAFLD groups (84 metabolites) than in the comparison between the normal-weight and overweight groups (48 metabolites), as illustrated in a Venn diagram (Figure S1C). In addition, most of the plasma triglyceride, diglyceride, and phosphatidylcholine levels were significantly elevated in the overweight group, irrespective of NAFLD presence, but they were also simultaneously selected as NAFLD markers (Figures S1D and S1E) which may act as concomitant variables. Considering these findings, we focused on a subpopulation with BMI z-scores > 1 (OC and ON groups) to identify promising candidates (Figure S1A, black arrow), then verified these in the normal-weight group.

Figure S2. Clinical characteristics of the study population according to the occurrence of obesity and NAFLD. Abbreviations: NAFLD, nonalcoholic fatty liver disease; BMI, body mass index; AST, aspartate aminotransferase; ALT, alanine aminotransferase; GGT, gamma-glutamyltransferase; ALP, alkaline phosphatase; HbA1c, Hemoglobin A1c; and HOMA-IR, homeostatic model assessment for insulin resistance.

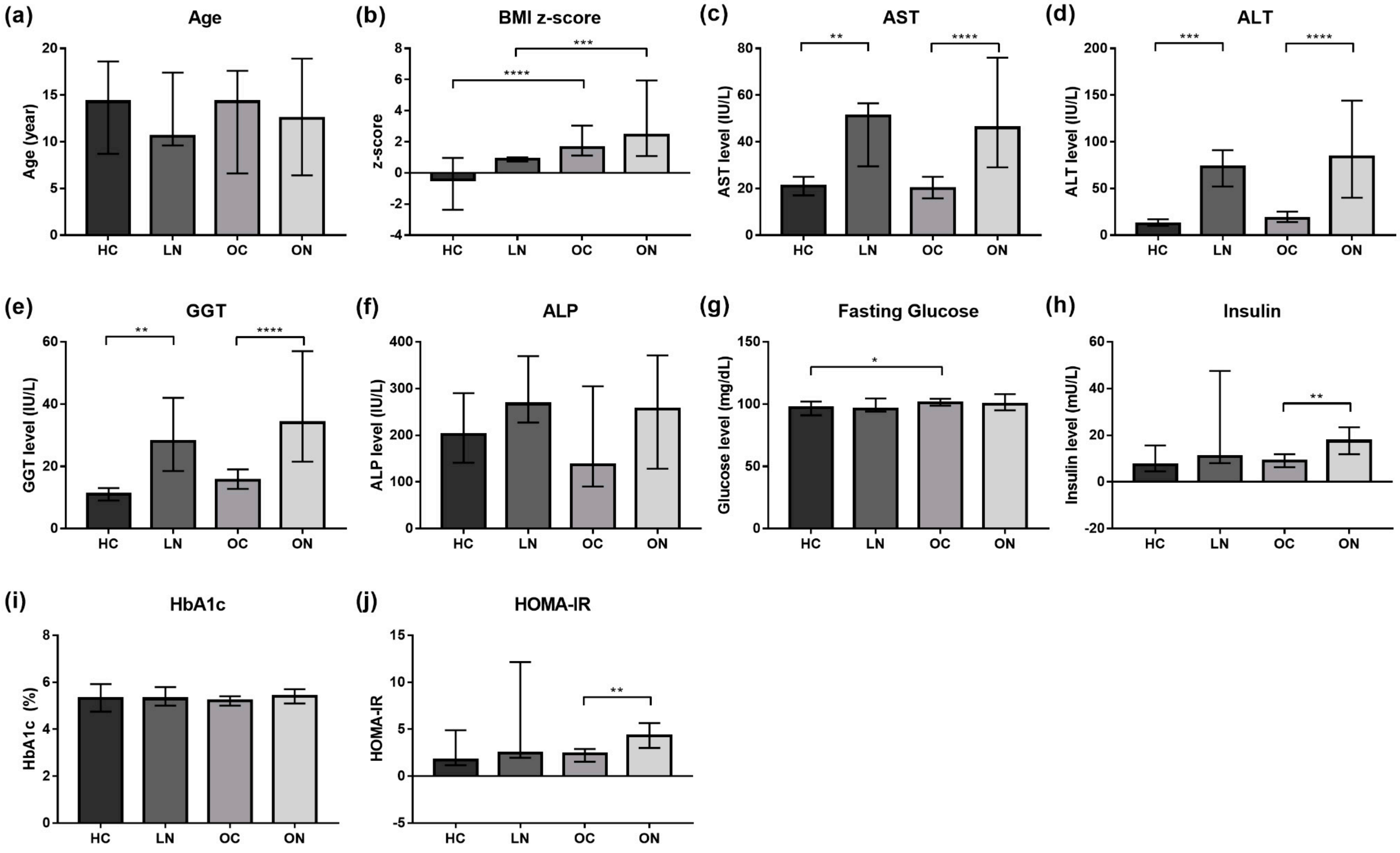

(a)-(b) Median bar charts with error bars indicating the range.

(c)-(j) Median bar charts with error bars indicating the interquartile range.
Significance by post-hoc Dunn’s multiple comparison test following Kruskal–Wallis test.
P-values: < 0.0332(*), < 0.0021 (**), < 0.0002 (***), < 0.0001 (****).

Figure S3. Pareto-scaled score plot of principal component analysis showing metabolomic distribution in the study population (HC, LN, OC, and ON).

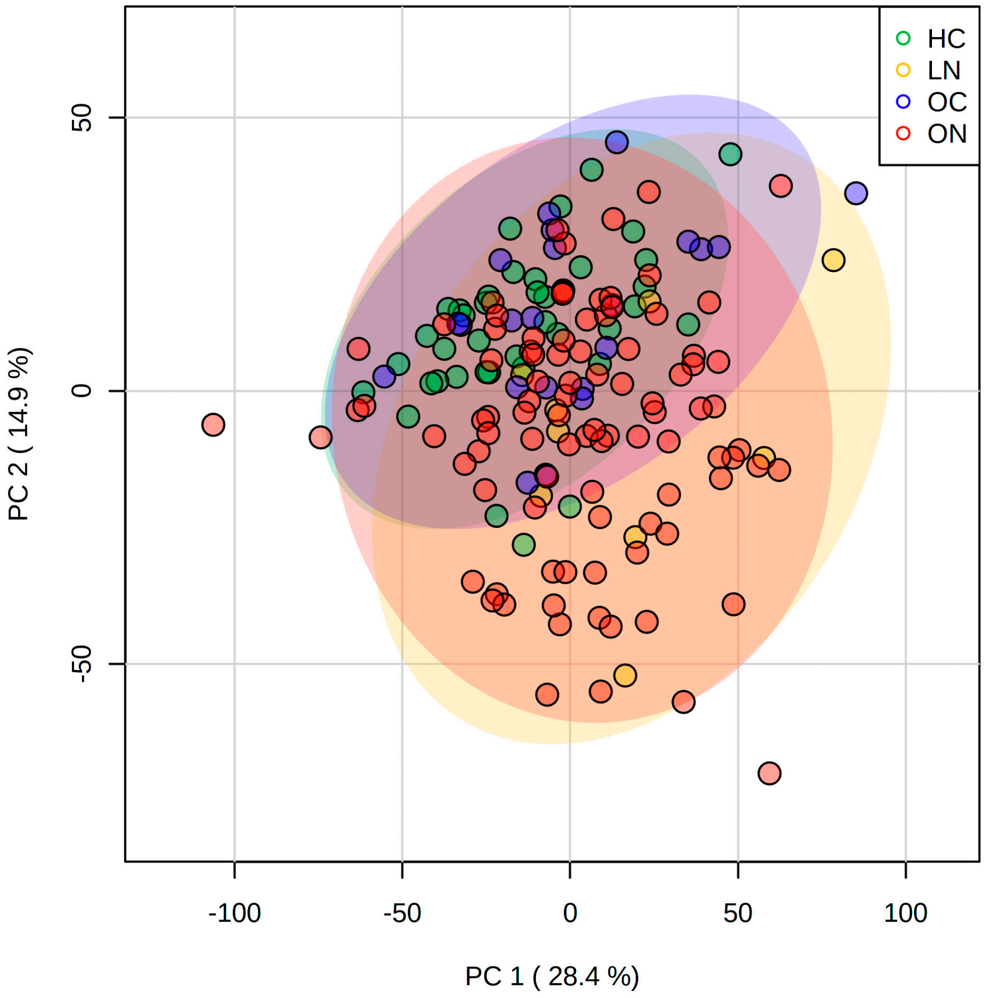

Figure S4. Spearman correlations between NAFLD-specific metabolic features and insulin resistance with $p < 0.05$, except Tyr, Lys, Gly, LPC (18:2), and PC-O (30:0). Abbreviations: Glu, glutamic acid; TG, triglyceride; DG, diglyceride; xLeu, sum of leucine and isoleucine levels; PC, phosphatidylcholine; SM, sphingomyelin; Ile, isoleucine; Val, valine; AC, acylcarnitine; Tyr, tyrosine; Lys, lysine; Gly, glycine; LPC, lysophosphatidylcholine; and PC-O, ether-linked phosphatidylcholine.

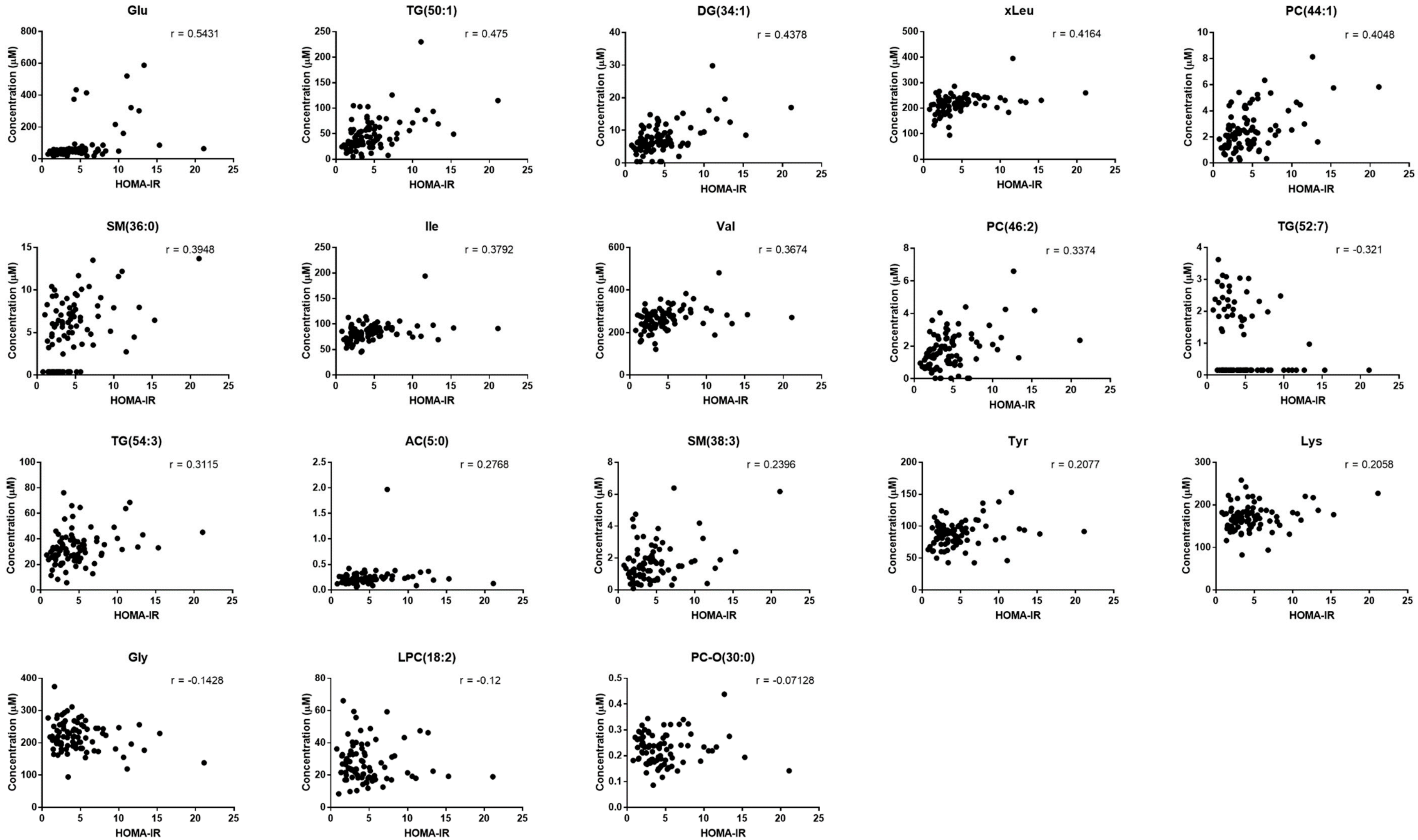
Glu
r = 0.5431
TG(50:1)
r = 0.475
DG(34:1)
r = 0.4378
xLeu
r = 0.4164
PC(44:1)
r = 0.4048
SM(36:0)
r = 0.3948
Ile
r = 0.3792
Val
r = 0.3674
PC(46:2)
r = 0.3374
TG(52:7)
r = -0.321
TG(54:3)
r = 0.3115
AC(5:0)
r = 0.2768
SM(38:3)
r = 0.2396
Tyr
r = 0.2077
Lys
r = 0.2058
Gly
r = -0.1428
LPC(18:2)
r = -0.12
PC-O(30:0)
r = -0.07128
Concentration (μM)
HOMA-IR
